# Exploring the diversity of biological processes regulated by glial cell line-derived neurotrophic factor, a pleiotropic molecule with therapeutic potential

**DOI:** 10.3389/fphys.2025.1618330

**Published:** 2025-06-26

**Authors:** Cristina Porcari, Stefano Cattaneo, Lucia Crippa, Michele Simonato, Barbara Bettegazzi

**Affiliations:** ^1^ Department of Neuroscience and Rehabilitation, Section of Pharmacology, University of Ferrara, Ferrara, Italy; ^2^ Division of Neuroscience, IRCCS Ospedale San Raffaele, Milan, Italy; ^3^ IRCCS Neuromed, Pozzili, Italy; ^4^ Università Vita-Salute San Raffaele, Milan, Italy

**Keywords:** GDNF (glial cell line-derived neurotrophic factor), GDNF secretion, GDNF signaling, astrocytic GDNF, GDNF physiological functions, GDNF role in the CNS

## Abstract

Glial cell line–derived neurotrophic factor (GDNF) is a potent trophic factor essential for neuronal survival and function. Encoded by the GDNF gene, its mature protein arises from specific post-translational modifications and is secreted through distinct isoform-dependent pathways. Once released, GDNF binds to its receptors, GFRα1 and RET, activating downstream signaling cascades that regulate cell growth, differentiation, and survival. In the central nervous system, GDNF exerts protective effects on dopaminergic neurons—highlighted in Parkinson’s disease research—and shows promise for modulating schizophrenia, depression, and addiction. Beyond dopaminergic pathways, GDNF influences synaptic plasticity in hippocampal neurons and supports GABAergic function. Glial cells also produce and respond to GDNF: astrocyte-derived GDNF can promote neuroprotection but also modulate microglial state and neuroinflammation. Other cell sources, such as pericytes and endothelial cells, contribute to GDNF levels, impacting blood-brain and blood-nerve barrier permeability. Peripherally, GDNF is critical for sympathetic and parasympathetic neuron development, somatic sensory neuron maintenance, and motor neuron reinnervation at the neuromuscular junction. Finally, GDNF has been recently implicated in tumour biology, underscoring its multifaceted role at the interface between beneficial and detrimental effects. Clinically, its therapeutic potential is being explored in different diseases, including neurodegenerative disorders and epilepsy. In this review, we will explore various aspects of GDNF biology and then focus our attention to the physiological mechanisms of GDNF-regulated processes in the central and peripheral nervous system, concluding with a brief perspective related to its therapeutic potential for central nervous system disorders. A deeper knowledge of the mechanisms regulating GDNF secretion and signaling, particularly the cellular source and the specificity of the GDNF-engaged intracellular signaling pathways, could be helpful to develop more precise therapeutic strategies for different CNS diseases.

## 1 Introduction

GDNF, the glial cell line-derived neurotrophic factor, is a neurotrophic factor involved in fundamental physiological processes in the central nervous system (CNS) and periphery. GDNF can elicit a variety of cellular responses, involved in several key processes, including neuronal survival, neurite outgrowth, and synaptogenesis. Beyond its role in development, GDNF exerts a significant neuroprotective role in different pathophysiological conditions, including neuroinflammatory and neurodegenerative diseases, but also some psychiatric disorders and addiction. The clinical potential of GDNF has been evident since its discovery in the nineties, nonetheless, the possibility to exploit this molecule in therapy has not yet fulfilled its promise. Indeed, the lack of knowledge of certain aspects of GDNF biology has slowed the progress of GDNF-based therapies. In this review, we focused on a wide assessment of the current knowledge about GDNF and its role in different physiological processes, highlighting the therapeutic opportunities arising from this knowledge.

### 1.1 The glial cell line–derived neurotrophic factor (GDNF)

The glial cell line-derived neurotrophic factor belongs to the GDNF family ligands (GFLs), which also include persephin, neurturin, and artemin (Airaksinen and Saarma, 2002). In physiological conditions, GDNF is expressed in soft tissue, testis, kidney, adrenal gland, parathyroid gland, placenta, gastrointestinal tract, spinal cord, and in different areas of the brain (The Human Protein Atlas - https://www.proteinatlas.org/ENSG00000168621-GDNF/tissue). Neurotrophic factors are proteins that support neuronal survival, cell proliferation, and differentiation. Additionally, they function as neurocytokines, facilitating communication between neurons and target tissues. GFLs members exert their function through binding as homodimers to the tyrosine kinase receptor Rearranged during Transfection (RET). In turn, RET activation occurs only if the GFL is bound to a member of the GDNF-family receptor-α (GFRα) receptors, which are anchored to the plasma membrane via a glycosyl phosphatidylinositol (GPI) linker, lacking transmembrane and intracellular domains (Airaksinen and Saarma, 2002). The intracellular pathways activated by all GFLs play a crucial role in promoting neuronal development and survival. Moreover, GDNF was initially isolated from rat glial cell lines and identified as a neurotrophic factor able to enhance dopamine uptake in midbrain dopaminergic neurons (Lin et al., 1993). The neuroprotective effect of GDNF has attracted significant interest from the scientific community for its potential use in treating neurodegenerative diseases (see below).

#### 1.1.1 GDNF, from the gene to the mature protein

**FIGURE 1:**
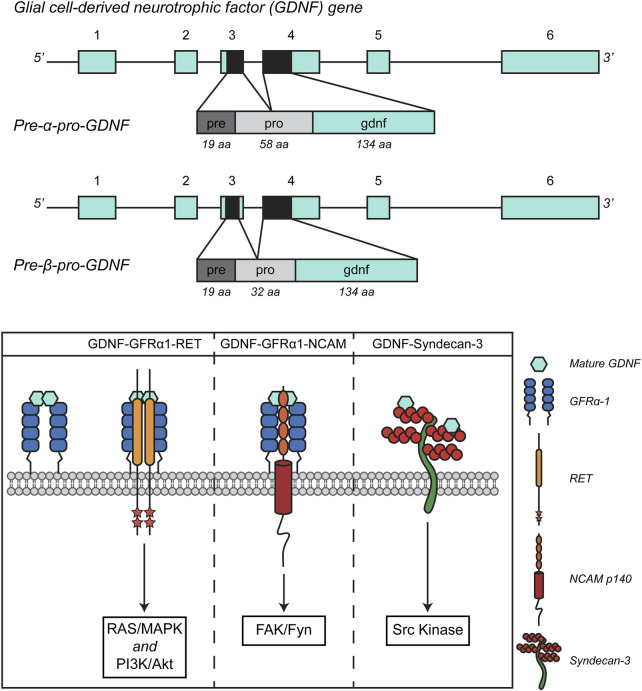
Schematic representation of GDNF splicing isoforms (A) and signaling pathways. Adapted from (Penttinen et al., 2018, Front. Neurol., CC BY 4.0).

The human GDNF gene is located on chromosome 5 (5p13.2), it is 12 kb-long and consists of six exons. An alternative splicing occurring at the level of the third exon gives rise to two conserved alternative isoforms: a full-length transcript, the pre-α-pro-GDNF, and a shorter version of the transcript, lacking 78 bp in the sequence encoding the pro region, named pre-β-pro-GDNF (Schaar et al., 1994; Cristina et al., 1995). This results in a 26 amino-acid difference in the pro region of the pre- α-pro-GDNF and pre- β-pro-GDNF (Figure 1 – upper panel). Both isoforms, however, lead to the same 134 amino-acid-long mature GDNF protein, since the proteolytic cleavage site that is crucial to obtain the mature protein is encoded in exon II, which is not affected by the alternative splicing (Cristina et al., 1995). The protein maturation process starts soon after synthesis, with the pre-sequence-mediated localization of the protein in the endoplasmic reticulum. The protein folds, disulfide (S-S) bonds are formed, and dimerization occurs. GDNF is also modified by N-linked glycosylation. After that, GDNF undergoes proteolytic processing into its mature form. The proteases that are involved in the processing of pro-GDNF to mature GDNF are furin, PACE4, and the following protein convertases: PC5A, PC5B, and PC7 (Lonka-Nevalaita et al., 2010). From a structural point of view, GDNF is characterized by a topological knot formed by three cysteines (Eigenbrot and Gerber, 1997), which makes it a distant member of the transforming growth factor-β (TGF-β) family. GDNF features two finger-like structures that play a role in its interaction with GFRα. The site where the protein undergoes post-translational modification through N-glycosylation is situated near one of these finger-like structures. Although this modification is not critical for receptor binding and activation, it is necessary for the proper folding and processing of GDNF in mammalian cells (Piccinini et al., 2013).

### 1.2 GDNF secretion

GDNF is a secreted protein, synthesized as a precursor protein (pre-pro-GDNF). Both the presence of the pro-domain and of the cysteine residues are important for secretion, that is strongly reduced in their absence (Oh-hashi et al., 2009; Piccinini et al., 2013). As mentioned before, in humans and rodents, GDNF is present in two splice isoforms, the pre-α-pro-GDNF and pre-β-pro-GDNF, that differ for a 26 amino-acid deletion in the pro domain of the β isoform. A study conducted in PC-6.3 cells showed distinct subcellular localizations for these two isoforms under normal conditions and after neuronal stimulation (Lonka-Nevalaita et al., 2010). Pre-α-pro-GDNF is primarily found in the Golgi complex and gradually progresses through the secretory pathway after stimulation. On the other hand, pre-β-pro-GDNF is mainly located in secretory vesicles and moves more quickly through the secretory pathway upon stimulation. The presence of both the α and β isoforms in secretory granules is confirmed by the co-localisation with the Rab3A and Rab27A markers. However, only pre-β-pro-GDNF shows strong co-localization with Secretogranin II, indicating its presence in the vesicles of the regulated secretory pathway, whereas pre-α-pro-GDNF shows less co-localization. Additionally, upon stimulation with potassium chloride, only the secretion of the mature GDNF form derived from the β precursor protein is increased (Lonka-Nevalaita et al., 2010). A potential reason for the difference between pre-α-pro-GDNF and pre-β-pro-GDNF could be that pre-α-pro-GDNF is less efficiently sorted into secretory granules at the trans-Golgi network, accounting for the higher presence of the α precursor in the Golgi region. Due to this inefficient sorting, some pre-α-pro-GDNF may also enter the constitutive secretion pathway.

### 1.3 GDNF signaling pathways

As previously mentioned, GFLs exert their trophic action through the activation of the RET receptor, but this interaction requires the presence of the co-receptor GFRα. Notably, there are four GFRα proteins capable of interacting with GFLs: GFRα1, GFRα2, GFRα3, and GFRα4, with GDNF binding preferentially to GFRα1 and with lower affinity to GFRα2, as demonstrated in mice studies (Cacalano et al., 1998). GDNF acts as a homodimer, stabilized by the formation of a disulfide bond. The GDNF-GFRα1 complex increases the affinity for the RET receptor and once bound, it triggers the dimerization of the RET receptor and the transphosphorylation of tyrosine residues in its intracellular portion, thereby activating intracellular signaling. Like other tyrosine-kinase receptors, the outcome of the GDNF-GFRα1-RET interaction is the engagement of signaling effectors that include the Ras/MAP kinase, PI3 kinase/AKT, and phospholipase C-γ (PLCγ) pathways (Ibanez, 2013).

Focusing on the subcellular organization of this interaction, GFRα1 is located on the outer leaflet of the plasma membrane via the GPI anchor, residing in detergent-insoluble, sphingolipid- and cholesterol-rich membrane microdomains known as lipid rafts (Tansey et al., 2000; Paratcha et al., 2001). In the context of GDNF signaling, it has been observed that, for efficient downstream signaling and maximal GDNF-mediated bioactivity, RET must be recruited to these lipid rafts by GFRα1. Additionally, activated RET interacts with Src family kynases (SFKs) only when it is localized within these microdomains (Tansey et al., 2000). In the absence of SFKs recruitment, a decrease in the activation of AKT and MAPK pathways is observed, highlighting the crucial role of Src activity for optimal GDNF-mediated neuronal survival and neurite outgrowth (Encinas et al., 2001). The intensity of RET engagement by GDNF can then be modulated by its localization inside or outside lipid rafts, as well as by the ability of GFRα to function in soluble form, the so-called “trans” signaling (Tansey et al., 2000; Paratcha et al., 2001).

The observation that GDNF and GFRα are widely expressed even in the absence of co-expressed RET suggests that the neurotrophic factor might exert its functions through alternative, RET-independent signaling systems (Trupp et al., 1997). GDNF can indeed interact with the Neural Cell Adhesion Molecule (NCAM), a cell surface glycoprotein crucial for neural development and plasticity. NCAM plays a key role in cell-cell adhesion, neuron growth, and migration during brain development. In addition to its ability to facilitate cellular interaction, NCAM, in conjunction with GFRα receptors, has been found to act as a RET-independent signaling receptor for GFLs. This interaction helps to explain the tissue distribution of GFRα proteins. Specifically, it has been demonstrated that GFRα can form a complex with NCAM, particularly with the p140NCAM isoform, at the cell surface. This interaction results in enhanced binding of GDNF and reduced homophilic interactions between NCAM molecules (Paratcha et al., 2003). The binding of GDNF activates Src-like kinase Fyn and focal adhesion kinase (FAK) in the cytoplasm, ultimately stimulating Schwann cell migration and promoting axonal growth in hippocampal and cortical neurons (Paratcha et al., 2003). GDNF can also act independently of both RET and NCAM. During development, GDNF and GFRα promote the differentiation of ventral precursors into GABAergic cells, enhancing their neuronal morphology and motility–see below (Pozas and Ibáñez, 2005). Moreover, all GFLs except persephin, when immobilized and bound to the extracellular matrix, interact with a transmembrane heparan sulphate proteoglycan named Syndecan-3. The interaction between GFLs and Syndecan-3 causes the activation of Src kinase and is crucial for promoting neurite outgrowth and cell spreading. Specifically, the GDNF-syndecan-3 interaction enhances the migration of cortical neurons (Bespalov et al., 2011). GDNF signaling pathways are summarized in Figure 1 – lower panel.

## 2 The role of GDNF in biological processes

GDNF is extensively distributed across both the CNS and peripheral tissues. In either physiological or pathological conditions, it can be produced and secreted by a variety of cell types, including neurons, glial cells such as astrocytes, Schwann cells, and oligodendrocytes, as well as by motor neurons and skeletal muscle cells (Henderson et al., 1994). Additionally, GDNF signaling is involved in the normal development and shaping of the ureteric bud in the kidneys, and it is also secreted by Sertoli cells within the testis, playing a role in self-renewal and proliferation of spermatogonial stem cell (Costantini, 2010). The crucial role of GDNF signaling in development is highlighted by the fact that mice deficient in RET, GDNF, or GFRα do not survive after birth, displaying kidney agenesis and a lack of many parasympathetic and enteric neurons. While mice lacking other GFLs or co-receptors are viable and fertile, the ones missing NRTN or GFRα2 show similar deficits in enteric and parasympathetic innervation.

### 2.1 Functions of GDNF in the CNS

#### 2.1.1 GDNF in neurons

GDNF expression increases during embryonic development of the CNS, decreasing instead in adulthood, when it remains restricted to specific brain areas such as the cortex, hippocampus, striatum, Substantia nigra, thalamus, cerebellum, and spinal cord (Hellmich et al., 1996; Mogi et al., 2001).

##### 2.1.1.1 Dopaminergic neurons

Since its initial isolation, GDNF has been regarded as a potential therapeutic neurotrophic factor playing a role in the development of Substantia nigra (SN) Dopaminergic (DA) neurons (Lin et al., 1993). In spite of its name, GDNF is not physiologically expressed in glial cells of the murine nervous system, but rather in neurons, particularly in parvalbumin-positive (PV+) interneurons, cholinergic and somatostatin-positive interneurons in the striatum, as demonstrated in transgenic mice with the lacZ cassette at the GDNF locus (Pascual et al., 2008; Gonzalez-Reyes et al., 2012; Hidalgo-Figueroa et al., 2012). The pre-α-pro-GDNF isoform appears to be the predominant isoform in the dopaminergic system, particularly in the striatum and substantia nigra (Airavaara et al., 2011). In healthy adult human brains, GDNF mRNA levels are typically low; however, they have been found to increase under pathological conditions such as Parkinson’s disease, not only in neurons but also in astrocytes, microglia, and macrophages (Nakagawa et al., 2005; Bäckman et al., 2006; Azevedo et al., 2020).

GDNF principal receptors, GFRα1 and RET, mRNA and protein have been found to be expressed in rodent midbrain DA neurons from early embryonic development through to adulthood (Trupp et al., 1997; Golden et al., 1999; Airaksinen and Saarma, 2002). Several studies on rodent models have investigated the role of GDNF, GFRα1, and RET in the midbrain dopaminergic system. In rat and mouse models of Parkinson’s disease, a transient increase followed by a decline in GFRα1 and RET mRNA levels was observed in the substantia nigra after 6-hydroxydopamine (6-OHDA) exposure (Marco et al., 2002). A similar reduction in RET receptor levels was reported in the striatum following 1-methyl-4-phenyl-1,2,3,6-tetrahydropyridine (MPTP) treatment (Hirata and Kiuchi, 2007), with both studies linking decreased GFRα1 and RET expression to the loss of tyrosine hydroxylase (TH)-positive dopaminergic neurons in the midbrain and their diminished innervation of the striatum (Hirata and Kiuchi, 2007). GDNF injection during the postnatal period has been shown to have a protective effect on the survival of substantia nigra (SN) dopaminergic neurons, while transgenic models overexpressing GDNF exhibit increased dopamine levels and striatal innervation (Burke, 2004; Kumar et al., 2015). However, GDNF and GFRα1 knockout mice die shortly after birth without evident alterations in the dopaminergic system. In contrast, RET deficiency leads to a progressive loss of SN dopaminergic neurons in aging mice (Kramer et al., 2007). Moreover, in a recently published study, the disruption of the *gdnf* gene in zebrafish embryos, utilizing CRISPR/Cas9 gene editing, resulted in a significant reduction (∼20%) of dopaminergic neurons in specific diencephalic clusters. This decrease was associated with altered expression of key transcription factors, including *otpb* and *lmx1b.1*, which are critical for dopaminergic neuron differentiation. Additionally, gdnf-deficient zebrafish exhibited impaired locomotor activity at 7 days post-fertilization and increased susceptibility to neurotoxic insults. These findings suggest that GDNF plays a conserved and essential role in the early development and functional maintenance of dopaminergic neurons (Wong et al., 2021). On the other hand, constitutive RET activation caused by a missense Meth918Thr mutation in the receptor results in an increased number of dopaminergic neurons in the SN and greater resistance to neurotoxins such as MPTP and 6-OHDA (Mijatovic et al., 2011). Moreover, conditional deletion studies of GDNF and RET have produced conflicting results regarding their necessity for neuronal survival in adulthood, suggesting the presence of compensatory mechanisms or alternative ligands (Pascual et al., 2008; Kopra et al., 2017). Several questions remain open, including the identity of the essential ligand for RET in the midbrain and the intracellular signaling pathways involved in neuronal survival.

##### 2.1.1.2 Hippocampal neurons

GDNF and its receptors, GFRα1 and NCAM, are expressed in hippocampal neurons during embryonic and early postnatal development, with GFRa1 localized at both pre- and post-synaptic sites, while NCAM is restricted to presynaptic terminals (Ledda, 2007). In hippocampal neuron cultures, GDNF enhances synapse formation, and the interaction of GFRα1-coated beads with neurons in the presence of soluble GDNF can induce ectopic presynaptic sites, demonstrating an instructive role of GDNF/GFRα1 signaling in synaptogenesis (Ledda, 2007), a process that partially relies on NCAM at presynaptic terminals. *In vivo*, mutant mice with reduced GDNF levels exhibit impaired presynaptic maturation and a decreased number of presynaptic sites during hippocampal development, further supporting the role of GDNF in synaptic assembly (Bonafina et al., 2019). Furthermore, utilizing crystallography and electron microscopy, it has been identified a decameric assembly comprising two GFRα1 pentamers bridged by five GDNF dimers. This configuration facilitates synaptic adhesion by forming complexes that bridge adjacent cell membranes. Further experiments demonstrated that the presence of the RET receptor and heparan sulfate can inhibit the formation of this adhesion complex by competing for the same binding interfaces (Bonafina et al., 2019). These findings suggest a dual role for GFRα1: promoting synaptic adhesion independently of RET and engaging in RET-mediated trophic signaling. This dual functionality provides insights into the molecular mechanisms underlying neuronal connectivity and the modulation of synaptic structures (Bonafina et al., 2019).

Additionally, GDNF/GFRα1 signaling is critical for the structural and functional integration of adult-born granule cells into preexisting hippocampal circuits. Conditional GFRα1 knockout mice display deficits in behavioural pattern separation, a function linked to adult neurogenesis. Notably, physical activity enhances GDNF expression in the dentate gyrus, promoting GFRα1-dependent CREB activation and dendritic maturation. These findings highlight GDNF/GFRα1 signaling as a key regulator of both developmental and adult hippocampal plasticity, orchestrating synaptogenesis and the incorporation of new neurons into functional circuits (Bonafina et al., 2019). Recently, it has also been demonstrated that elevated levels of GDNF enhance GABAergic inhibitory inputs onto pyramidal neurons in the CA1 region of the hippocampus (Mikroulis et al., 2022). This effect is mediated through the activation of the RET receptor pathway, facilitated by the co-receptor GFRα1. Notably, the other GDNF receptors, namely as NCAM or Syndecan3, are not implicated in this process. The study also demonstrated similar enhancements in inhibitory synaptic transmission in human hippocampal slices obtained from epilepsy patients. These findings suggest that GDNF’s ability to strengthen inhibitory signaling may contribute to its observed seizure-suppressant effects in various epilepsy models (Paolone et al., 2019; Wahlberg et al., 2020; Mikroulis et al., 2022).

##### 2.1.1.3 Serotonergic neurons

Recent research has revealed significant interactions of GDNF with the serotonergic (5-HT) system. Serotonergic neurons of the raphe nuclei express GDNF receptors, particularly GFRα1 and RET, suggesting direct responsiveness to GDNF (Okaty et al., 2015; 2020; Huang et al., 2019; Ren et al., 2019). The effect of GDNF on the brain 5-HT system is controversial, with some studies showing positive effects on brain 5-HT (Pertusa et al., 2008; Naumenko et al., 2013), and others reporting little or no effect (Hudson et al., 1995; Mijatovic et al., 2007). On the other hand, serotonin increases GDNF expression and secretion from C6 rat glioma cells, acting predominantly via 5-HT2A receptors (Hisaoka et al., 2004; Tsuchioka et al., 2008). While moderate increases in GDNF enhance serotonin neuron number, serotonergic gene expression, and 5-HT levels, excessive GDNF leads to a decrease in serotonergic neurons differentiation (Menegola et al., 2004). This nonlinear relationship may explain previous conflicting reports on GDNF’s effects on the serotonergic system.

Human data with antidepressants provide indirect evidence of a connection between GDNF and the serotonergic system. Acute or chronic administration of antidepressants increasing the levels of synaptic serotonin (such as tricyclic antidepressants, tetracyclic antidepressants, and serotonin-selective reuptake inhibitors) is indeed able to increase GDNF expression both in cell culture (Mercier et al., 2004; Hisaoka et al., 2007; Golan et al., 2011; Kajitani et al., 2012; Hisaoka-Nakashima et al., 2015; 2019; Abe et al., 2019) and in serum of patients with depression (Zhang et al., 2008). Preclinical data show that animals exposed to chronic unpredictable stress exhibit depression-like behavior and decreased GDNF expression in the hippocampus, that is reverted by chronic tricyclic antidepressant treatment (Uchida et al., 2011; Liu Q. et al., 2012). In addition, GDNF may be decreased in the peripheral blood of patients with major depressive disorder (Takebayashi et al., 2006; Lin and Tseng, 2015; Sharma et al., 2016).

These findings suggest that the modulation of GDNF production may be a component of the therapeutic effect of antidepressants and that the fine-tuning of the serotonergic system by GDNF could be implied in the mechanisms of development of neuropsychiatric disorders. This nuanced relationship positions GDNF as a key neurotrophic factor beyond its classical dopaminergic role, critically involved in serotonergic system regulation (Popova et al., 2017).

##### 2.1.1.4 GDNF and neurogenesis

Glial cell line-derived neurotrophic factor (GDNF) plays a multifaceted role in neurogenesis, influencing neural progenitor proliferation, migration, and differentiation across developmental and adult stages. GDNF has been recognized as a chemoattractant and differentiation signal for neuronal precursors, particularly in the subventricular zone and rostral migratory stream (Paratcha et al., 2006), where it modulates key signaling pathways, including RET/GFRα1 and PI3K/Akt, enhancing neurogenic output and synaptic integration (Airaksinen and Saarma, 2002).

In the forebrain, inhibitory GABAergic interneurons originate in the ventral telencephalon and migrate tangentially to reach the developing cortex, hippocampus, and olfactory bulb (Bartolini et al., 2013). The ganglionic eminences serve as temporary neurogenic regions, with the medial and caudal ganglionic eminences (MGE and CGE) generating most cortical GABAergic neurons, whereas the lateral ganglionic eminence (LGE) primarily contributes interneurons to the olfactory bulb. Both GDNF and its co-receptor GFRα1 are expressed in the MGE and along the migratory routes of GABAergic neurons (Pozas and Ibáñez, 2005), where GDNF facilitates differentiation and functions as a chemoattractant (Pozas and Ibáñez, 2005; Paratcha et al., 2006). These effects depend on GFRα1 but not on NCAM or RET, and the addition of soluble GFRα1 to MGE cultures enhances differentiation and migration even in cells lacking endogenous GFRα1 (Perrinjaquet et al., 2011). Moreover, syndecan-3 has been proposed as an alternative GDNF receptor in MGE-derived GABAergic neurons, independently of GFRα1 (Bespalov et al., 2011). Mice deficient in GFRα1 exhibit reduced migration of GABAergic neurons, leading to a lower number of inhibitory neurons in the cortex and hippocampus at birth (Pozas and Ibáñez, 2005), as well as disrupted integration of parvalbumin-expressing neurons and altered social behaviors linked to increased cortical excitability (Canty et al., 2009), a phenomenon consistent with certain autism models (Tabuchi et al., 2007). GFRα1 signaling is also crucial for olfactory system development, as its loss results in deficits in multiple GABAergic interneuron populations in the olfactory bulb, along with impairments in neurogenesis, migration, and sensory axon growth (Marks et al., 2012).

In stroke models, direct GDNF infusion significantly increases neurogenesis in the striatum (Kobayashi et al., 2006), while in the hippocampus, GDNF and its receptor GFRα1 are essential for proper integration of adult-born granule neurons (Bonafina et al., 2019). Following ischemia and traumatic brain injury, expression of various growth factors is increased and modulates neurogenesis, NSPC biology, and striatum connectivity (Christie and Turnley, 2013; Bacigaluppi et al., 2020; Butti et al., 2022). More recently, the neurogenic efficacy of GDNF has been harnessed through biomaterial-based delivery systems that enhance spinal cord repair and remyelination (Liu et al., 2024). Transplanting mesenchymal stem cells engineered to overexpress GDNF significantly improves neuroregenerative outcomes following ischemic or traumatic injury (Salgado et al., 2015; Chiavellini et al., 2022). This therapeutic strategy offers promising avenues in treating neurodegenerative disorders highlighting GDNF not only as a neuroprotective factor but also as a potent pro-neurogenic agent with promising translational potential in regenerative therapies.

##### 2.1.1.5 GDNF and neuroplasticity

Neuroplasticity, the ability of the central nervous system to promote neurogenesis and renew connections, is influenced by different psychological, environmental and physiological factors, often involving the synthesis and secretion of neurotrophins (Kempermann et al., 2018).

For example, several recent studies highlight that engaging in regular physical exercise, whether aerobic or strength-based, can enhance cognitive function and promote neuroplasticity through different mechanisms (De Sousa Fernandes et al., 2020). Aerobic exercise primarily boosts glutamatergic signaling and neurotrophic factors like BDNF and CREB, while resistance training engages pathways involving PKCα and inflammatory cytokines, while it has been shown that both kinds of exercise share as a common outcome the upregulation of GDNF in plantaris myofibers (Gyorkos and Spitsbergen, 2014; Rabelo et al., 2017; Vilela et al., 2017). GDNF likely plays a central role in mediating the cognitive benefits of exercise by promoting hippocampal remodelling and resilience to age-related neural decline (Vilela et al., 2017). In young adult mice, voluntary exercise increases levels of GDNF and BDNF in the dentate gyrus (DG) (Cotman and Berchtold, 2002; Farmer et al., 2004). This upregulation correlates with enhanced dendritic growth and complexity in newly generated granule cells, suggesting a role for these factors in mediating activity-dependent neuronal integration. Notably, the effects of exercise on granule cells maturation seems to be dependent on GFRα1, the co-receptor for GDNF. Mechanistically, GDNF/GFRα1 signaling may act through the activation of the transcription factor CREB, which is known to regulate activity-dependent dendritic development (Jagasia et al., 2009). *In vitro* stimulation of DG-derived neural stem cell cultures with GDNF lead to phosphorylation of both CREB and Erk1/2, confirming that GDNF directly activates this signaling cascade in differentiating neurons (Bonafina et al., 2019).

#### 2.1.2 GDNF in glial cells

As discussed above, GDNF is almost exclusively expressed by neurons in physiological conditions in the CNS (Pochon et al., 1997; Hidalgo-Figueroa et al., 2012), although its transcript can also be found in other cell types (Schaar et al., 1994). However, in various diseases, its expression in the brain changes over time, and other cell populations may become new reservoirs of GDNF production. Thus, in the following section, we will focus on the role of glia-derived GDNF.

##### 2.1.2.1 GDNF in astrocytes

A growing body of evidence is pointing at a fundamental role of astrocyte-derived GDNF, which contributes to neuroprotection by modulating synaptic function, reducing oxidative stress, and promoting neuronal survival. Studies on primary astrocytic culture highlighted that inflammatory stimuli distinctly regulate GDNF and Neuregulin-1 (NRG-1) in rodent astrocytes and microglia, with LPS treatment significantly increasing GDNF expression in astrocytes (Bresjanac and Antauer, 2000; Iravani et al., 2012; Kronenberg et al., 2019; Merienne et al., 2019). However, inflammatory stimuli are not the only ones able to impact GDNF expression. In an *in vitro* model of ischemia, utilizing neuron-glia and astrocyte cortical cultures subjected to oxygen and glucose deprivation, astrocyte-GDNF emerged as one of the released factors that mediated neuroprotection, elicited by high-frequency repetitive magnetic stimulation (Gava-Junior et al., 2023). Cinnamon and its metabolite sodium benzoate (NaB) can upregulate GDNF in human astrocytes. Oral administration of NaB and cinnamon increased astrocytic expression of GDNF in a model of PD *in vivo*, conferring neuroprotection of TH neurons of the *Substantia Nigra Pars Compacta*. However, this effect was absent in astrocyte-specific GDNF knockout mice (GDNF∆astro) (Patel et al., 2019). In another PD model, Gemfibrozil, a lipid-lowering drug approved by the FDA, has been shown to stimulate astrocytic GDNF, protecting dopaminergic neurons, through a PPARα-dependent pathway. Interestingly, Gemfibrozil was not effective in GDNF∆astro mice lacking GDNF, specifically in astrocytes (Gottschalk et al., 2021). Reactive astrocytes in Parkinson’s disease models also exhibit neurotrophic functions, with Nestin-positive astrocytes expressing GDNF (Chen et al., 2006). Furthermore, astrocytic GDNF mitigates cognitive decline post-anesthesia by improving hippocampal synaptic plasticity (Lin et al., 2024).

Conversely, exposure to di-(2-ethylhexyl) phthalate (DEHP), an environmental endocrine-disrupting compound used in food packages, medical devices, office supplies, and children’s toys, reduces the secretion of GDNF, interfering with the estrogen pathway, by downregulating the ERK/c-fos signaling in astrocytes (Wang et al., 2021). Excessive GDNF levels have been linked to astrocyte proliferation and potential gliomagenesis via the GFRα1/RET/MAPK/pCREB/LOXL2 axis (Wang et al., 2022). Finally, chronic overexpression of GDNF in brain astrocytes in a transgenic mouse model appears to have a detrimental influence on nigrostriatal dopamine metabolism and neurotransmission (Sotoyama et al., 2017).

##### 2.1.2.2 GDNF in microglial cells

GDNF produced by activated microglia and macrophages can aid in the repair of CNS injuries. In a model of spinal cord injury, LPS-induced macrophage activation enhanced GDNF expression at lesion sites, promoting functional recovery of the spinal cord by sustaining a neurotrophic environment, while mitigating oxidative stress (Hashimoto et al., 2005). GDNF mRNA was also upregulated a few hours post-injury, in a model of mechanical injury in the mouse striatum, with brain macrophages as the primary source, establishing a critical link between GDNF expression and dopaminergic neuron protection (Liberatore et al., 1997). Microglia, which persist long after injury, contribute significantly to neurotrophic support, predominantly secreting GDNF and BDNF. This immune-mediated neurotrophic environment facilitates dopaminergic axonal sprouting and tissue repair, particularly in neurodegenerative contexts such as PD (Batchelor et al., 1999; 2002). Notably, sprouting dopaminergic fibers associate closely with neurotrophic factor-expressing microglia, exploiting them as structural support to navigate toward lesion edges (Batchelor et al., 2002). NG2-positive and Iba1-positive cells in the substantia nigra express GDNF and it has been shown that they are localized close to surviving TH-positive neurons in the *Substantia Nigra Pars Compacta*, suggesting a neuroprotective role for these cells in dopaminergic neuron survival (Kitamura et al., 2010). Moreover, experiments on BV2 cells have shown that GDNF exerts an anti-inflammatory effect by decreasing the release of pro-inflammatory cytokines such as TNF-α, TGF-β, IL-1β, and IL-12β in a model of inflammation induced by amyloid beta. This study highlights the involvement of the Hippo/YAP signaling pathway in this process (Qing et al., 2020). These findings collectively highlight the interplay between immune cells and neurotrophic factors in CNS repair.

##### 2.1.2.3 GDNF and neuroinflammation

GDNF has long been recognized for its pivotal role in promoting axonal growth and neuronal regeneration, particularly within the peripheral nervous system (Lee et al., 2016). However, emerging evidence underscores its potent anti-inflammatory effects, highlighting its critical role in modulating neuroinflammation in several neurological diseases (Bido et al., 2024; Dingledine et al., 2024). Neuroinflammation is a complex process involving immune cell activation, vascular modulation, and alterations of resident cells, namely astrocytes and microglia. It can have both beneficial and detrimental effects on brain pathologies (Simonato et al., 2006; DiSabato et al., 2016).

A key aspect of this response is mediated by pattern recognition receptors (PRRs), which detect pathogen- and damage-associated molecular patterns (PAMPs and DAMPs), leading to immune cell activation (Iravani et al., 2012; Singh et al., 2022). Recent studies have shown that neurotrophic factors, including BDNF, NGF, and GDNF, play a crucial role in regulating neuroinflammatory pathways (Rocha et al., 2012; Guarino et al., 2022). Among these, GDNF is expressed by both astrocytes and microglia in pathological conditions, with astrocyte-secreted GDNF exerting a strong inhibitory effect on microglial activation, thereby mitigating neuroinflammation. GDNF can modulate the activation of microglia after Zymosan A (yeast-derived immune stimulant) treatment. It has been shown that the effect is dependent on GFRα1 and neutralization of GDNF or GFRα1, as well as GDNF silencing in astrocyte cultures, abolishes its regulatory effects, confirming that GDNF binding to the microglial GFRα1 receptor initiates intracellular signaling cascades responsible for suppressing microglial activation (Rocha et al., 2012).

In hippocampal astrocytes, GDNF/GFRα1 signaling contributes to neuroprotection by regulating immune responses. The upregulation of GDNF and its receptor GFRα-1 in hippocampal neurons and astrocytes enhances resilience against thrombin-induced neurotoxicity, known mechanisms to activate microglia, and induce neuronal death (Yun et al., 2020). Recently, it has been highlighted that astrocytic Sterile Alpha and TIR Motif Containing 1 (SARM1), which plays a critical role in axonal degeneration and inflammation in Multiple Sclerosis (MS), promotes neuroinflammation and axonal demyelination by suppressing GDNF expression. Moreover, pharmacological reduction of GDNF worsened disease progression, reinforcing its neuroprotective function and therapeutic potential in MS (Jin et al., 2022). Beyond microglial regulation, GDNF modulates immune responses through GFL receptors, which are also expressed in immune cells. Activation of these receptors leads to the suppression of immune cell activity and the regulation of pro-inflammatory mediator release (Vargas-Leal et al., 2005).

#### 2.1.3 GDNF from other cell sources in the CNS

Besides the already discussed cell types, GDNF has been identified in less abundant cellular components, in particular endothelial cells and pericytes, where it can act as a modulator of Blood-Brain-Barrier (BBB) and Blood-Nerve-Barrier (BNB) (Sharma et al., 2022). The BBB is a protective membrane that regulates molecular exchange between the bloodstream and neural tissue. Brain endothelial cells strictly limit the entry of molecules, particularly harmful ones, into the brain parenchyma. Indeed, a major challenge in CNS drug development is determining whether drug candidates can effectively cross the BBB. In recent years, several BBB and BNB models have been proposed, and Trans Endothelial Electrical Resistance (TEER) has been used as a key measure of barrier integrity, with values of 500 Ω × cm^2^ or higher indicating an intact BBB and values of 150 Ω × cm^2^ or higher indicating an intact BNB (Yosef and Ubogu, 2013). Studies have shown that GDNF strengthens the BBB by increasing tight junction protein expression, such as claudin-5, leading to elevated TEER values in various models, including porcine BBB and human brain microvascular endothelial cells (Igarashi et al., 1999; Shimizu et al., 2011; Kanjanasirirat et al., 2024). Similar effects were also demonstrated for BNB, where it was shown that MAPK signaling was essential for GDNF-mediated BNB TEER increase (Dong and Ubogu, 2018).

Finally, oligodendrocytes have been shown to produce and secrete GDNF. In particular, differentiated oligodendrocytes were identified as a source of GDNF, which can activate distinct intracellular pathways in neurons (Wilkins et al., 2003). In particular, decreased release of GDNF by these cells has been linked with neurodegeneration in a model of Multiple system atrophy (Ubhi et al., 2010).

### 2.2 Functions of GDNF in the peripheral nervous system

Initially identified for its role in dopaminergic neuron survival, GDNF is a crucial neurotrophic factor that supports the survival, maintenance, and regeneration of neurons in the peripheral nervous system (PNS). GDNF influences the development and function of peripheral neurons, including sensory, motor, and autonomic neuronal cells. It is produced by Schwann cells, muscle cells, and target tissues, creating a supportive microenvironment for nerve regeneration following injury. GDNF enhances axonal growth, promotes remyelination, and prevents neuronal apoptosis.

#### 2.2.1 Development of sympathetic and parasympathetic neurons

Four ganglia in the cranial region host postganglionic parasympathetic neurons: the ciliary, sphenopalatine, submandibular, and otic ganglia. In newborn mice lacking RET, GFRα1, or GDNF, the otic and sphenopalatine ganglia are not present, indicating that the GDNF signaling through the GFRα1-RET receptor complex is critical for the development of these parasympathetic neurons during embryogenesis. Indeed, at embryonic day 12, these ganglia are already missing, and their neuronal precursors exhibit defects in migration and proliferation. Therefore, GDNF signaling through GFRα1–RET is necessary for the migration and proliferation of specific parasympathetic neuronal precursors in the early stages of embryonic development (Enomoto et al., 2000).

#### 2.2.2 Somatic sensory neurons

Although *in vitro* studies showed that GFLs can support specific subpopulations of primary sensory neurons, their precise physiological functions *in vivo* remain largely unclear. Before birth, the survival of numerous primary sensory neurons in the petrosal ganglion relies on target-derived GDNF and BDNF (Erickson et al., 2001). These visceral chemoafferent neurons, which innervate the carotid body, play a role in the regulation of breathing. Indeed, mice lacking GDNF or RET exhibit respiratory disturbances, and mutations in these genes have been associated with congenital central hypoventilation syndrome (Manié et al., 2001).

#### 2.2.3 Enteric neurons

Enteric neurons and ganglia originate from vagal and sacral neural crest cells, which migrate from the neural tube to the gut wall. Within the gut, they initially move in a rostrocaudal direction before transitioning from superficial to deeper layers. Following migration, these cells proliferate and differentiate to form the enteric plexus. In this context, GDNF/RET signaling is fundamental for the migration, proliferation, and survival of enteric neural crest cells (ENCCs) during the development of the enteric nervous system (Obermayr et al., 2013; Nagy and Goldstein, 2017). GDNF is secreted by mesodermal cells of the gut mesenchyme (Young et al., 2001), as well as by intestinal smooth muscle and epithelial cells in some pathological conditions (Xiao et al., 2014; Meir et al., 2015; Le Berre-Scoul et al., 2017). RET is expressed by ENCCs, while its co-receptor GFRα1 is required for efficient signaling. Studies in mice have demonstrated that the loss of function in GDNF, RET, or GFRα1 results in the complete absence of the enteric nervous system, underscoring the critical role of this pathway in gut innervation (Schuchardt et al., 1994; Moore et al., 1996; Cacalano et al., 1998). Experimental studies have shown that GDNF functions as a potent chemoattractant, reliably directing ENCC migration *in vitro* and in *ex vivo* explants (Natarajan et al., 2002; Wang et al., 2010). Overexpression or systemic administration of GDNF enhances ENCC proliferation and migration, increasing their numbers within the gut. However, while GDNF plays a crucial role in supporting the survival and neurogenesis of neuronal-fated ENCCs, it does not independently induce neural fate determination. Instead, it acts as a permissive factor, allowing the differentiation of ENCCs into specific neuronal subtypes at appropriate developmental stages (Wang et al., 2010). Recent studies also showed that GDNF supports intestinal barrier maturation and protects against inflammation-induced damage in inflammatory bowel disease (IBD). This finding highlights the role of enteric glial cells as a key source of GDNF, showing that its secretion enhances barrier function and prevents inflammatory breakdown, underscoring the importance of these cells in intestinal homeostasis (Meir et al., 2015; 2021). Collectively, these findings highlight the indispensable role of GDNF/RET signaling in enteric nervous system development, regulating ENCC migration, proliferation, and differentiation while ensuring the formation of functionally diverse neuronal populations.

#### 2.2.4 Motor neurons

GDNF plays a crucial role in motor neuron survival and development (Henderson et al., 1994; Oppenheim et al., 1995). While it promotes motor neuron viability *in vitro*, studies in knockout mice have demonstrated that the absence of GDNF or its receptor RET results in a significant loss of lumbar spinal motor neurons at birth, particularly affecting γ-motor neurons that innervate intrafusal muscle spindles (Moore et al., 1996; Sánchez et al., 1996; Gould et al., 2008; Bonanomi et al., 2012). This indicates that GDNF-dependent RET activation is essential for early motor neuron development, acting through the co-receptor GFRα1 (Gould et al., 2008). However, RET signaling does not appear necessary for motor neuron survival in adulthood but contributes to neuromuscular junction (NMJ) maintenance and muscle innervation. Evidence suggests that amyloid precursor protein (APP)-regulated GDNF expression is crucial for NMJ integrity and muscle function (Stanga et al., 2016).

Interestingly, altered GDNF levels have been observed in muscle biopsies and cerebrospinal fluid samples from ALS patients, suggesting its involvement in the disease (Yamamoto et al., 1999; Stanga et al., 2018). While its precise role in the pathology remains unclear, the GDNF/RET pathway may contribute to neuroprotection, as indicated by studies on the ALS drug Edaravone, which appears to exert its neuroprotective effects through this signaling axis (Li et al., 2022). Conversely, recent findings suggest that inhibiting RET tyrosine kinase activity could enhance retrograde transport in motor neurons (Rhymes et al., 2022), and its interaction with p75 may even promote apoptosis under certain conditions (Donnelly et al., 2018), highlighting the complexity of RET signaling in motor neuron health and disease.

#### 2.2.5 GDNF delivery for regenerative medicine

GDNF plays a critical neurotrophic role in the regeneration of the peripheral nervous system, especially in supporting motor neuron survival, promoting axonal outgrowth, and enabling target muscle reinnervation after injury. GDNF delivery has been shown to protect motor neurons from apoptosis, stimulate robust axonal regeneration, and improve reinnervation of muscle targets, ultimately restoring neuromuscular function (Cintron-Colon et al., 2022). Recent studies highlight that timed delivery of GDNF enhances long-distance axonal regeneration and functional recovery in severe injury models like ventral root avulsion (Eggers et al., 2020a; 2020b). Sustained GDNF expression via viral vectors or hydrogels promotes neuromuscular junction repair and muscle fiber preservation, especially when delivered locally to the nerve or muscle (Kokai et al., 2011; Cintron-Colon et al., 2022). Furthermore, GDNF combined with biomaterial scaffolds such as nerve conduits or engineered Schwann cell grafts has shown enhanced reinnervation efficiency and reduced denervation-induced muscle atrophy (Zhang et al., 2009; Carvalho et al., 2021), although some contrasting results on the GDNF release and dosage are emerging (Kong et al., 2021). Altogether, these findings establish GDNF as a pivotal factor in peripheral nerve repair strategies, supporting both the neural and the muscular components of regeneration.

### 2.3 Function of GDNF outside the nervous system

Outside the nervous system, GDNF is essential for kidney morphogenesis by mediating reciprocal inductive signaling between the nephrogenic mesenchyme and the ureteric bud (Sariola and Saarma, 2003). GDNF, expressed in the mesenchyme, binds to RET and GFRα1 on the ureteric bud, promoting its branching and nephron formation (Suvanto et al., 1996; Sainio et al., 1997). Knockout studies of GDNF, RET, or GFRα1 result in severe renal defects, highlighting their critical role (Pichel et al., 1996; Saarma and Sariola, 1999). Factors such as heparan sulfate proteoglycans and transcription factors like Pax2 and Eya1 further regulate GDNF expression and function in kidney development (Brophy et al., 2001).

GDNF is secreted by Sertoli cells and plays a crucial role in regulating spermatogenesis through paracrine signaling. RET and GFRα1 are expressed in undifferentiated spermatogonia, which include spermatogenic stem cells. GDNF dosage is critical for stem cell balance: reduced levels lead to excessive differentiation and depletion, while overexpression causes undifferentiated spermatogonia clustering and infertility (reviewed in (Parekh et al., 2019).

#### 2.3.1 GDNF and tumors

GDNF and its receptors play a crucial role in various cancers by influencing cell proliferation, migration, and invasion. In neuroendocrine tumors, GDNF is highly expressed in growth hormone-secreting pituitary adenomas but absent in most other pituitary tumors, suggesting a potential link to growth hormone signaling (Japo´n et al., 2002). RET activation by GDNF regulates somatotroph populations via the p53 apoptotic pathway, with Pit-1 transcription factor mediating this effect (Shewchuk et al., 2006; Cañibano et al., 2007). In pancreatic cancer, GDNF and its receptors are widely expressed, promoting tumor proliferation, invasion, and perineural infiltration through integrin β1 and matrix metalloprotease-9 (MMP-9) upregulation (Okada et al., 2003; Liu H. et al., 2012). Similarly, gliomas exhibit elevated GDNF levels, which enhance migration via the MAPK and JNK pathways and confer chemoresistance, while Growth Arrest Specific 1, a protein that is structurally homologous with the GFRα receptors, blocks this process by inhibiting RET signaling (Song and Moon, 2006; Domínguez-Monzón et al., 2009; Ng et al., 2009). In colorectal cancer, GDNF activates the RET/GFRα1 complex, enhancing β1 integrin expression and increasing VEGF-VEGFR-mediated migration through p38, PI3K/Akt, and HIF1α pathways (Huang et al., 2014). Breast cancer cells also express RET and GFRα1, responding to both autocrine and paracrine GDNF signaling (Neve et al., 2006; Esseghir et al., 2007; Kang et al., 2009). Testicular cancer is associated with GDNF overexpression, promoting invasive seminoma behavior (Sariola and Meng 2003; Ferranti et al., 2012). In melanoma, GDNF-driven RET activation correlates with malignancy, significantly enhancing proliferation and invasion through phosphorylated Tyr905 in human melanoma cells (Kato et al., 1998; 2007; Narita et al., 2009; Ohshima et al., 2010).

Collectively, these findings underscore the oncogenic potential of GDNF signaling across multiple cancer types. The GDNF-RET axis functions as a double-edged sword that requires precise regulation to achieve therapeutic benefits. In certain oncological diseases, inhibiting this axis may be advantageous, while enhancing it could be vital for the survival of neurons in various neurological disorders. In the final chapter, we will concentrate on the latter therapeutic approach.

## 3 Challenges and future directions

### 3.1 Future directions in the elucidation of GDNF biology

**FIGURE 2:**
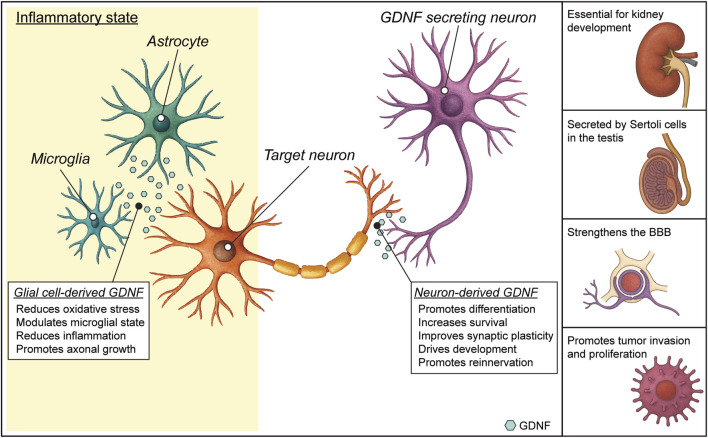
Glial cell line-derived neurotrophic factor (GDNF) functions. In the healthy nervous system, GDNF expression is mainly neuronal. The engagement of GDNF receptors in the neuronal membrane by GDNF binding triggers an intracellular signaling cascade that promotes different effects, such as survival and differentiation. In diseased states, GDNF can also be expressed by glial cells. Glial GDNF expression can promote neuroprotection, neuronal survival and modulate astrocyte and microglial activation, but sustained GDNF overexpression can lead to adverse effects. Outside the CNS (lateral panel), GDNF has fundamental roles in kidney morphogenesis, spermatogenesis in testis and maintenance of the blood brain barrier. GDNF can also have a role in inducing proliferation of certain tumors (see text).

Looking at the complex biology of GDNF (Figure 2), there are still several areas where our understanding remains incomplete. While we know some transcription factors [e.g., EGR1, CREB - (Liu et al., 2020; Marks et al., 2023)] that can induce GDNF expression, the full regulatory network controlling its expression in different tissues and under different physiological or pathological conditions is not well mapped and we still do not completely understand how GDNF expression is modulated in response to injury, inflammation, or neurodegeneration. From the signaling point of view, the biological relevance and downstream effects of the engagement of RET-independent pathways are still being explored, to define if they are compensatory, redundant or used to generate specialized signals.

The same can be said regarding context-dependent signaling, with specific responses, occurring most likely due to differences in receptor expression, co-signaling molecules, or epigenetic state of the cells, that still need to be clarified. For example, GDNF role in normal homeostasis of mature neurons, especially in the adult brain and peripheral autonomic system, is not well defined, while its role in pathology has been more characterized. GDNF interacts or overlaps with other neurotrophic factors like NGF, BDNF and Neurturin, but the functional cross-talk, synergy, or competition among these factors is still not fully understood. Finally, the mechanisms of secretion, diffusion, and gradient formation of GDNF *in vivo* remain technically hard to measure and model. Understanding GDNF distribution, degradation and turnover will be essential for both biology and therapeutic design.

### 3.2 Clinical potential of GDNF

Since its identification, GDNF, along with other GFLs, has sparked significant interest in the scientific community for its potential as a therapeutic agent in treating various neurological disorders. Multiple pre-clinical studies demonstrated that GDNF delivery supports the long-term motor neuron survival and axon regeneration following peripheral nerve injury in both newborn and adult mice (Hottinger et al., 2000; Eggers et al., 2019; Cintron-Colon et al., 2022). Additionally, GDNF has been found to have a potent analgesic effect in neuropathic pain models (Boucher et al., 2000) and to facilitate sensory axon regeneration into the spinal cord after dorsal root injury, thereby restoring sensory function (Ramer et al., 2000).

GDNF has also emerged as a potential antiepileptic candidate. GDNF and its receptor are expressed in the pyramidal and granule cells of the hippocampus, and a link has been found between the neurotrophic factor levels and epilepsy, since locally increasing GDNF levels in the temporal lobe can suppress epileptic activity (Kanter-Schlifke et al., 2007; Nanobashvili et al., 2019; Paolone et al., 2019).

In experimental models of focal ischemia, administering exogenous GDNF before or immediately after anoxia has been shown to reduce ischemic brain injury. Specifically, GDNF appears to mitigate excitotoxic neuronal death through an ERK-dependent pathway, making early administration crucial in the treatment of stroke (Wang et al., 1997).

The involvement of GDNF in regulating dopaminergic neuronal plasticity has shown promise in influencing the biochemical adaptation processes and the rewarding effects associated with drug addiction, suggesting its potential application in the treatment of substance or alcohol abuse (Messer et al., 2000; Ford et al., 2023).

Given its vital role in promoting the survival of dopaminergic neurons, GDNF has also demonstrated its ability to prevent neurotoxin-induced death of dopamine neurons and to facilitate functional recovery in various animal models of Parkinson’s disease (Kearns and Gash, 1995; Tomac et al., 1995; Airaksinen and Saarma, 2002; Barker et al., 2020). These findings paved the way for the transition to clinical studies. Initial Phase I and II trials using direct intraventricular or intraputaminal infusion of GDNF (Kordower et al., 1999; Gill et al., 2003; Nutt et al., 2003; Love et al., 2005; Slevin et al., 2005) showed promising motor improvements and good safety profiles. However, a larger placebo-controlled Phase II trial (Lang et al., 2006) failed to show significant clinical benefit, raising concerns about inconsistent delivery and variable efficacy. More recent efforts in PD have focused on using convection-enhanced delivery (CED) systems (Barua et al., 2013; Taylor et al., 2013; Whone et al., 2019) and gene therapy vectors [e.g., AAV2-GDNF - (Rocco et al., 2022; Heiss et al., 2024)] to improve distribution and achieve sustained expression in the putamen of treated patients. These trials have shown better target coverage and encouraging biomarker responses (increased [18F]-DOPA uptake), but clinical improvements (and particularly systemic motor score improvements) remain modest or variable (Barker et al., 2020), underscoring the limitations that still need to be overcome to fully exploit GDNF therapeutic potential.

### 3.3 Current limitations of GDNF as a therapeutic molecule

Despite encouraging preclinical results, clinical translation remains complex due to issues related to optimal dosing, delivery methods, and long-term safety. Nonetheless, GDNF continues to be a promising candidate for the development of novel neurorestorative therapies. One of the primary challenges is effective delivery to target tissues, particularly the central nervous system, due to the BBB. Direct intracerebral administration, while bypassing the BBB, is highly invasive and may result in uneven distribution and local tissue damage. Furthermore, one of the biggest challenges is indeed optimization of the dose of GDNF, which is complicated by its narrow therapeutic window. Indeed, low doses may be insufficient to elicit a neuroprotective or neurorestorative effect, while high doses can lead to adverse physiological changes. For instance, in preclinical models, excessive GDNF has been shown to cause ectopic or abnormal sprouting of dopaminergic fibres, especially in the striatum, which may disrupt normal circuitry (Georgievska, 2002; Marshall, 2023). It can also downregulate tyrosine hydroxylase, a key enzyme in dopamine synthesis, thereby paradoxically impairing dopaminergic signaling over time. Short half-life and rapid degradation in the extracellular space further limit its efficacy when delivered exogenously. In addition, variability in patient response and limited efficacy observed in clinical trials in Parkinson’s disease highlight the need for improved delivery platforms, such as gene therapy or controlled-release systems. Finally, concerns regarding long-term safety, immune responses, and potential off-target effects pose additional hurdles that must be addressed before GDNF can be widely adopted in therapeutic settings.

Another layer of complexity is the temporal aspect: GDNF’s effects may vary depending on the stage of disease progression, meaning that timing and duration of treatment are just as critical as dose magnitude. Chronic overexpression—such as in some preclinical gene therapy approaches—may lead to long-term dysregulation of neuronal homeostasis or immune activation.

Finally, inter-individual variability—due to differences in GDNF receptor (RET and GFRα1) expression levels, regional pathology, or genetic background—further complicates standardized dosing protocols, making personalized approaches potentially necessary for safe and effective use.

## 4 Conclusion

GDNF has long been studied for its neuroprotective effects, however, it also has broader physiological roles that still need elucidation. GDNF is indeed critical for the development, survival, and maintenance of dopaminergic, sympathetic, parasympathetic, and enteric neurons, and it plays essential roles in organ systems such as the kidney, testis, and gastrointestinal tract. Its involvement in tissue regeneration and modulation of inflammatory responses underscores its broader impact on homeostasis. Finally, the diversity of GDNF signaling pathways makes it a rich model for exploring ligand-receptor dynamics, signal integration, and cell-type specificity. In this review we explored GDNF’s endogenous functions—beyond its therapeutic potential—providing insight into its fundamental role as a neurotrophic factor in neural and non-neural systems, reframing GDNF as a multifaceted regulator of physiological function, rather than solely as a neurotrophic therapeutic.

## Glossary

GDNF: Glial cell line-derived neurotrophic factor
GFLs: GDNF Family Ligands
RET: Rearranged during Transfection
GFRα1: GDNF Family Receptor alpha 1
GPI: Glycosylphosphatidylinositol
TGF-β: Transforming Growth Factor beta
PC5A/5B/7, PACE4, Furin: Proprotein Convertases
pre-α/β-pro-GDNF: Alternative precursor isoforms
PI3K: Phosphoinositide 3-kinase
AKT: Protein kinase B
MAPK: Mitogen-Activated Protein Kinase
JNK: c-Jun N-terminal Kinase
Erk1/2: Extracellular signal-regulated kinases 1/2
CREB: cAMP Response Element-Binding Protein
PLCγ: Phospholipase C gamma
SFKs: Src Family Kinases
NCAM: Neural Cell Adhesion Molecule
FAK: Focal Adhesion Kinase
PC-6.3: PC-6.3 cells
Rab3A/Rab27A: Ras-related proteins
ER: Endoplasmic Reticulum
SN: Substantia Nigra
DA neurons: Dopaminergic neurons
TH: Tyrosine hydroxylase
PV+: Parvalbumin-positive
6-OHDA: 6-Hydroxydopamine
MPTP: 1-Methyl-4-phenyl-1,2,3,6-tetrahydropyridine
CNS: Central Nervous System
PNS: Peripheral Nervous System
ECM: Extracellular Matrix
BDNF: Brain-Derived Neurotrophic Factor
NGF: Nerve Growth Factor
CREB: cAMP response element-binding protein
PKCα: Protein Kinase C alpha
DG: Dentate Gyrus
NRG-1: Neuregulin-1
OGD: Oxygen-glucose deprivation
NaB: Sodium Benzoate
PD: Parkinson’s Disease
PPARα: Peroxisome proliferator-activated receptor α
DEHP: Di-(2-ethylhexyl) phthalate
LOXL2: Lysyl oxidase-like 2
NG2: Neural/glial antigen 2
Iba1: Ionized calcium-binding adapter molecule 1
TNF-α: Tumor Necrosis Factor alpha
IL-1β: Interleukin-1 beta
IL-12β: Interleukin-12 beta
YAP: Yes-associated protein
PRRs: Pattern Recognition Receptors
PAMPs: Pathogen-associated molecular patterns
DAMPs: Damage-associated molecular patterns
MS: Multiple Sclerosis
SARM1: Sterile Alpha and TIR Motif Containing 1
BV2: BV2 cell line
Zymosan A: Yeast-derived immune stimulant
